# Serratia marcescens Endocarditis in Prosthetic Valves

**DOI:** 10.7759/cureus.48957

**Published:** 2023-11-17

**Authors:** Asma Jamil, Hari Sharma, Ruhma Ali, Alan Klukowicz, Abu Taha, Amy Paige, Sharath Bellary, Abbas Ahmed, Richard Miller, Priscilla Chow

**Affiliations:** 1 Pulmonary and Critical Care Medicine, Saint Michael's Medical Center, Newark, USA; 2 Ophthalmology, University of San Francisco, San Francisco, USA

**Keywords:** methicillin-resistant staphylococcus aureus (mrsa), serratia marcescens endocarditis, iv: intravenous, hepatitis c (hcv) infection, hiv, white blood count (wbc), uti: urinary tract infection, extended spectrum beta-lactamase (esbl)

## Abstract

Serratia marcescens endocarditis is a rare occurrence. We describe a case of Serratia endocarditis in a patient with a prosthetic valve. The clinical course was complicated by widespread embolic phenomena causing stroke, gangrene of extremities, and septic emboli to the lungs, spleen, and eyes. She was not considered suitable for surgery due to severe consumptive coagulopathy and thrombocytopenia in the setting of widespread emboli. The patient was transitioned to do not resuscitate status and discharged to a long-term care facility with a grave prognosis explained to the family.

## Introduction

Serratia marcescens (S. marcescens) resulting in endocarditis has been described in roughly 70 case reports. A review of the literature showed only one case report of Serratia endocarditis in the prosthetic tricuspid valve due to active intravenous (IV) drug use [1].

IV drug use remains the most common cause of community-acquired infection, while nosocomial sources remain the most common cause of hospital-acquired/nursing home-acquired infection. 

Unfortunately, most of the time, patients who have already undergone valve replacement do poorly if they get recurrent infections. They are not considered a good surgical candidate for repeat surgery if needed due to the high-risk behavior of continuous IV drug use.

We present a similar case with a worse outcome. Our patient suffered from disseminated emboli of endocarditis affecting her prosthetic tricuspid and mitral valves. She was not considered stable enough to undergo any surgical intervention. Our patient's code status was made do-not-resuscitate, she underwent tracheostomy and percutaneous esophago-gastrostomy (PEG) tube placement, and she was discharged to long-term acute care (LTAC).

## Case presentation

A 29-year-old, white female with a past medical history of polysubstance abuse including active IV drug abuse of heroin and smoking cocaine, untreated hepatitis C, and history of extended-spectrum beta-lactamase (ESBL) urinary tract infection presented from a nursing home with a complaint of fever and generalized body pain.

Patient's surgical history was significant for open heart surgery for valve repair three years ago due to methicillin-resistant Staphylococcus aureus (MRSA) endocarditis involving tricuspid and mitral valves. She underwent radical mitral valve reconstruction with posterior leaflet resection, annuloplasty (Cosgrove-Edwards) along with radical tricuspid valve resection with posterior leaflet resection, plication, and ring annuloplasty (Cosgrove-Edwards).

The patient has been doing IV heroin drug abuse since the age of 16. She continued IV heroin even after valve replacement surgery. She used to be homeless before getting sick.

Approximately two months ago, she was admitted to a different hospital for the second time with MRSA endocarditis complicated by an embolic right posterior temporal stroke. The patient responded to telavancin and vancomycin. The patient was sent to a different nursing home where she spent 24 hours and went back to a different emergency room due to hearing loss. The hearing impairment was attributed to vancomycin. Her antibiotic was changed to telavancin and cefepime was continued for another two weeks. The patient was sent to a nursing home for rehabilitation as a result of left-sided weakness due to the embolic stroke. She had completed the six-week course of antibiotics for endocarditis at this point.

About two weeks later, the patient presented to our hospital with fever, generalized body pain, and leukocytosis on labs. The patient denied chest pain, shortness of breath, nausea, vomiting, and diarrhea. The patient also denied any frequency, dysuria, hematuria, focal weakness, or headache.

Physical exam was significant for mild abdominal tenderness, and bilateral costovertebral angle tenderness. Heart sounds were normal with no audible murmur. Bilateral extremities showed ecchymosis from thrombocytopenia and frequent blood draws. No signs of splinter hemorrhages or Janeway lesions. Peripheries were warm.

Vitals on arrival revealed a temperature of 101.8 F, a blood pressure of 98/66 mmHg, a pulse of 113 beats per minute, and oxygen saturation of 98% on room air. 

The patient got around 3 L of fluid bolus. After that, patient's blood pressure improved. Initial labs upon presentation are outlined in Tables 1, 2.

**Table 1:** Labs upon initial presentation. Wbc: White blood count; Hgb: hemoglobin; BUN: blood urea nitrogen

Labs		Reference value
Wbc	17.9	4.4-11 x 1000/microlit
Hgb	9.5	12.0-15.5 g/dl
Platelets	98	150-450 x 1000/microlit
Neutrophils	15.9	1.7- 7 x 1000/microlit
Lymphocyte	1.2	0.9-2.9 x 1000/microlit
Sodium	128	136-145 mmol/L
Potassium	5.3	3.5-5.3 mmol/L
Chloride	94	98-110mmol/L
Bicarbonate	21	20-31 mmol/L
BUN	27	6-24mg/dl
Creatinine	1.2	0.5-1 mg/dl

**Table 2:** Labs upon initial presentation. AST: Serum aspartate transferase; ALT: serum alanine transferase; ALP: alkaline phosphatase; LDH: lactate dehydrogenase; BNP: brain-like natriuretic peptide; APTT: activated prothrombin time; PT: prothrombin time; INR: international normalized ratio

Labs		Reference value
Glucose	100	70-140 mg/dl
Lactate	7.2	0-2 mmol/L
Troponin	222	<14ng/L
AST	97	10-36 U/L
ALT	47	6-29 U/L
ALP	133	33-115 U/L
CRP	22	0.3-1 mg/dl
Procalcitonin	19	0-0.5ng/ml
albumin	2.9	3.6-5.1g/dl
LDH	472	122-222 U/L
BNP	1833	<100 pg/ml
APTT	31.9	26.9-36.3 seconds
INR	1.63	0.9-1.1
PTT	19.3	10.6-12.9 seconds
Fibrinogen	160	200-393mg/dl

Contrast-enhanced CT scan (CECT) of the abdomen and pelvis only showed wall edema of the sigmoid colon, rectum, and hepatosplenomegaly.

**Figure 1:**
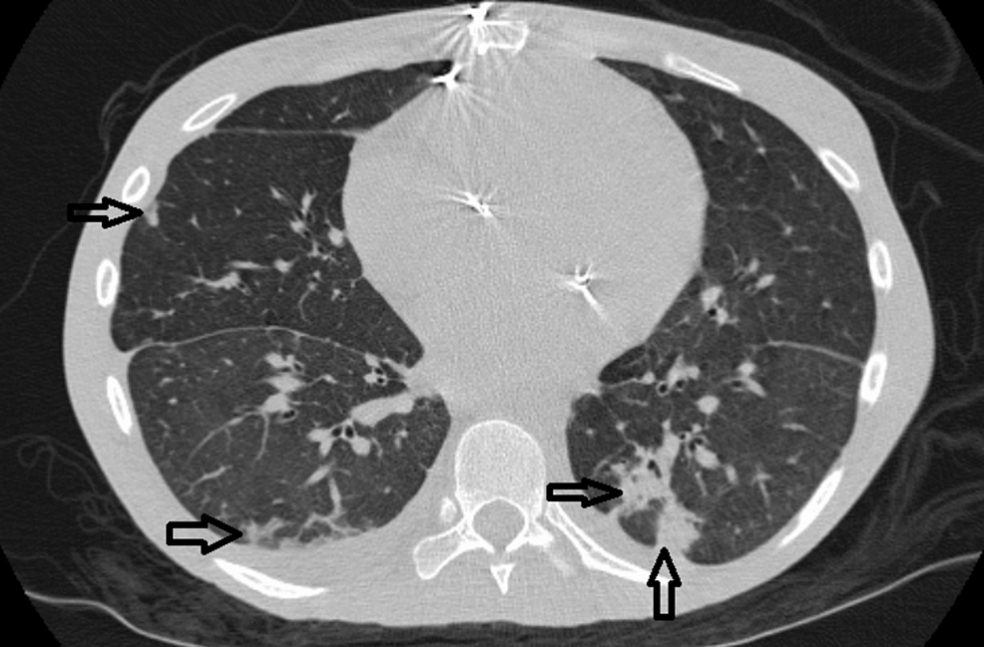
CT chest without contrast: Arrows indicate suspected peripheral wedge-shaped infarcts from endocarditis.

CT chest without contrast revealed non-specific patchy bilateral infiltrates with suspected wedge-shaped infarction from previous endocarditis episodes as outlined in Figure 1.

Ultrasound (US) abdomen revealed hepatomegaly, gallbladder stones, and sludge with suspected pericholecystic fluid with gallbladder wall thickening.

The patient was started on daptomycin 8 milligram/kilogram (mg/kg) and 2 gram(g) every 12 hours of ceftriaxone. Creatine phosphokinase (CPK) during treatment remained low. For her withdrawals she was managed on methadone, and later on buprenorphine. 

The next day, lactate improved from 7 to 4 mg/dl but leukocytosis persisted at 20 x 10 ^3^ cmm/dl. Liver function tests continued to get worse. 

Antibiotics later changed to piperacillin-tazobactam 3.375 mg every six hours, then to meropenem 1 g every eight hours due to clinical deterioration. Daptomycin was stopped early due to thrombocytopenia. About four days later, septic shock developed, and the patient was started on vasopressors.

The blood culture growth showed 4 out of 4 bottles of S. marcescens in the blood susceptible to ertapenem, gentamicin, and meropenem and resistant to piperacillin-tazobactam and ceftriaxone.

A repeat blood culture showed persistent bacteremia. Leukocytosis continued to get worse from 17 x 10 ^3^ to 26 x 10 ^3^ k and then 28 x 10^3^ cmm/dl. The patient's hemoglobin stayed around 8.2 to 10 mg /dl. There was a decrease in the platelet count from 98,000 on admission to 25,000 cmm/dl. 

**Figure 2:**
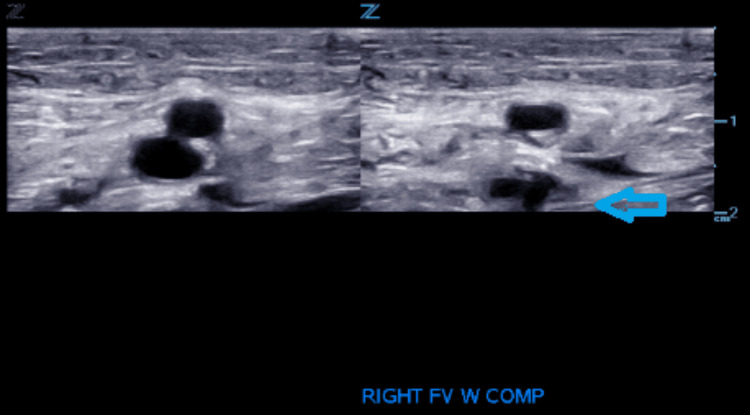
Venous duplex ultrasound: cross-sectional view: right femoral vein non-compressibility indicates thrombus presence.

**Figure 3:**
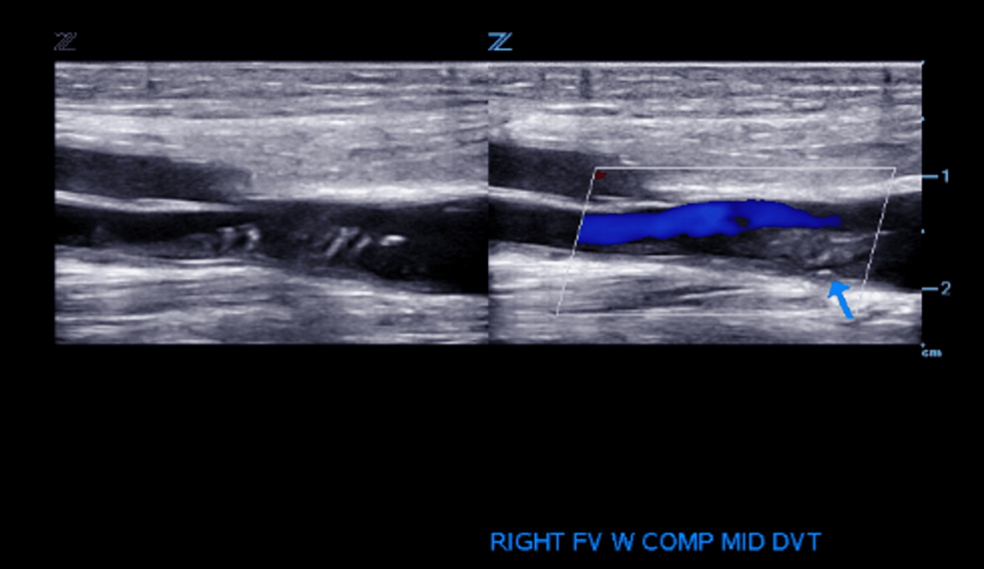
Venous duplex US: longitudinal view: thrombus in the right femoral vein.

**Figure 4:**
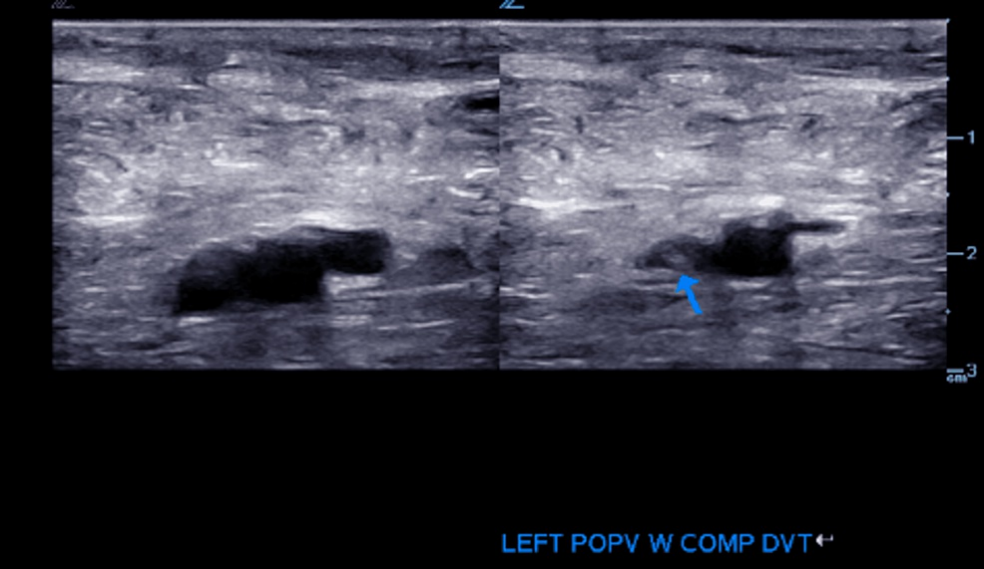
Venous duplex ultrasound: cross-sectional view: left popliteal vein with thrombus.

**Figure 5:**
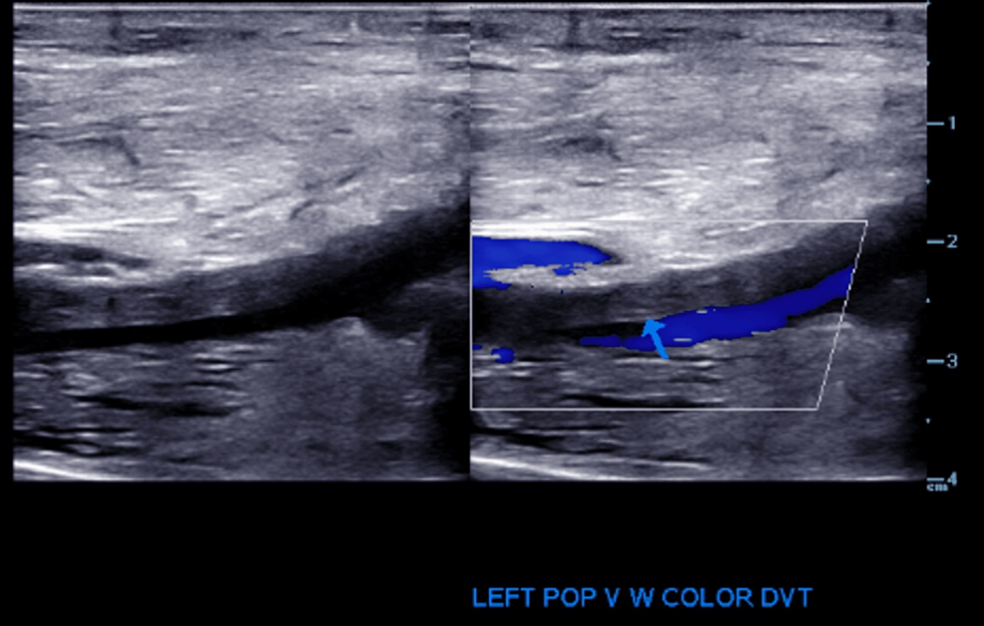
Venous duplex ultrasound: longitudinal view: left popliteal vein showing thrombus.

During hospitalization, cyanotic discoloration of the patient's feet was noted. US of the bilateral lower extremity showed deep vein thrombosis (DVT) in the right femoral vein and left popliteal vein as outlined in Figures 2-5.

An inferior vena cava (IVC) filter was placed due to the inability to do therapeutic anticoagulation for DVT, secondary to severe thrombocytopenia. The drop in platelets was attributed to S. marcescens sepsis and consumptive coagulopathy from both DVT and sepsis.

Echocardiogram done showed an ejection fraction (EF) of 55%, severely enlarged right ventricle (RV), severe RV dysfunction, 1.5 x 1.3 cm vegetation on the anterior mitral leaflet, and 1.2 x 0.5 cm tricuspid vegetation. Echo clips show vegetation on valves (Videos 1-3).

**Video 1:** Echo: Parasternal long axis view: arrow pointing toward vegetation on the anterior mitral valve leaflet.

**Video 2:** Echo: Parasternal long axis view: arrow pointing toward vegetation on the anterior mitral valve leaflet.

**Video 3:** Echo: 4 chamber view: arrow pointing towards vegetation on anterior mitral valve leaflet and tricuspid valve leaflet.

The patient was now noted to have physical exam changes with a new holosystolic murmur at the mitral valve and appearance of splinter hemorrhages in fingernails and Janeway lesions in hands. She was transferred to a higher level of care due to the possibility that she may need extracorporeal membrane oxygenation and nitric oxide during repeat open heart surgery. 

Before transfer, she was unarousable with GCS 4, she was intubated for airway protection. CT head done without contrast initially showed no acute changes. After being transferred to a higher level, repeat imaging revealed an acute ischemic stroke. 

CT angiogram of the head and neck showed an abrupt cut-off of the proximal left internal carotid artery. Complete non-opacification of the left internal carotid artery (ICA) indicated occlusion at the level of the carotid terminus. Also, there was acute left basal ganglia infarct related to an intraluminal thrombus of the left middle cerebral artery (MCA) 1 segment. The patient underwent emergent neuro intervention with mechanical thrombectomy in the left ICA/MCA with complete restoration of blood flow.

Later, magnetic resonance imaging (MRI) of brain showed evolving acute infarcts in the left basal ganglia and left parahippocampal regions with hemorrhagic transformation. There was partial effacement of the left lateral ventricle and a stable 2 mm rightward midline shift. Additional punctate acute infarcts in bilateral cerebral hemispheres were present.

Etiology was thought to be septic emboli from infective endocarditis, as the patient relapsed. Repeat imaging CT chest, abdomen, and pelvis with contrast revealed acute pulmonary emboli along with left lower lobe pulmonary infarct, occluded right external iliac artery, and proximal left external iliac artery. It also showed an enlarged spleen with multiple peripheral low attenuating foci suspicious for infarct. Hepatomegaly with heterogeneous parenchymal enhancement, moderate volume ascites, marked mesenteric edema, and anasarca was present as well.

Based on CT scan findings, all four extremity US was done. US of upper extremities revealed occlusive deep venous thrombi within the left mid and distal subclavian vein as well as within the left axillary vein. There was DVT in bilateral lower extremities as evidenced in the above images.

She has had multiple septic embolic events to the brain, lungs, and spleen likely from both right and left-sided vegetations on her heart valves. In addition to her bacteremia, she also had fungemia and trichomonas/mycoplasma infection. She was treated with intravenous micafungin, then fluconazole, metronidazole, and merropenem (2g every 8 hours) and later continued on IV fluconazole (200 mg every 24 hours), oral vancomycin (125 mg daily), and IV ciprofloxacin (400 mg every 12 hours). Blood culture later became negative. Ophthalmology evaluation revealed diffuse Roth spots but no evidence of endophthalmitis.

For DVT, she was treated with argatroban and later with apixaban after her thrombocytopenia stabilized. Later she developed dry gangrene of bilateral feet, surgical amputation was deferred at a later time due to lack of demarcation. 

She underwent tracheostomy and PEG tube placement. The endocarditis team noted the patient to have incurable disease with recurrent bacteremia but also has prohibitive risk factors for surgery such as poor neurologic status, new ongoing embolic strokes, profound thrombocytopenia, and coagulopathy. In lieu of all that, she was not considered a surgical candidate. She was transferred to a step-down level of care so that she can continue to receive treatment. She was transitioned to a long-term care facility after multiple goals of care conversations with the family. She was discharged on ciprofloxacin, fluconazole, and vancomycin to complete the six-week course.

## Discussion

S. marcescens is a non-lactose fermenting, aerobic (facultative anaerobe), oxidase-negative, gram-negative bacillus organism ubiquitous to the environment and is found in soil, water, and plants. It is not a part of normal human flora [2].

In a case report reviewing cases from 1969-1974, 19 cases of Serratia have been reported for the first time in literature [3].

The pathogenicity of S.marcescens has been attributed to its fimbriae-shaped structures and biofilm formation that allow adherence to valves, instruments, and indwelling catheters. It is also able to survive and grow under extreme conditions, including disinfectants, double distilled water antiseptics, and extreme temperatures, allowing it to cause infection successfully in healthcare settings [4].

Case reports describing Serratia outbreaks from bronchoscopes, contaminated chloroxylenol soap, surgical razors, contaminated respirators/nebulizer machines, stethoscopes, vials, and other fomites have been extensively described in the literature [5-13]. Invasive procedures such as surgery (arthrocentesis), use of contaminated vials, use of single-use medication vials on multiple patients, use of a common syringe for multiple medications/patients, and use of multidose vials have been associated with outbreaks of nosocomial infections. In those cases, rapid clearance of Serratia is noticed instead of long courses [4-13].

Outbreaks in neonatal intensive care units (ICU) have been described in multiple countries and in case reports [14-16]. Serratia infections can be classified as community-acquired or nosocomial [4]. Community-acquired infections are almost exclusively seen in intravenous drug users [16-18]. Hospital-acquired infections by Serratia have been attributed to a lack of adequate sterile conditions and hand hygiene [4].

Patients most at risk are those in ICU with large central venous access and those treated with broad-spectrum antimicrobial drugs [4]. Comorbidities found in patients developing endocarditis included structure disease of the heart e.g. coronary artery disease, aortic stenosis, patent foramen ovale, ventricular septal defects, and arrhythmia such as atrioventricular block. Systemic conditions associated with Serratia endocarditis described in the meta-analysis included pyelonephritis, chronic kidney disease, renal failure, nosocomial infection, cirrhosis, SLE, obesity, non-Hodgkin's lymphoma, recent history of dental work, HIV, HBV, and HCV [19].

Case reports have described Serratia endocarditis and its sequelae in the form of valvular abscess formation, embolic phenomena, aorto-atrial fistula formation, ventricular septal defects, septic arthritis, and meningitis [20,21].

In a meta-analysis by Lutman et al., S. marcescens endocarditis was reported in a total of 64 cases between 1964 and 2020. Patients' ages ranged from neonate (<4 weeks of age) to 85 years old with a mean age of 42 years. Sex distribution revealed that about 28 (43.8%) were females and 35 (54.7%) males. Echocardiogram findings were reported in 26.6% of cases. The location of vegetation was described in 71.9% of cases. The mitral valve was the most common source of vegetation followed by the aortic valve, tricuspid valve, and pulmonic valve. Six cases have multiple valve involvement concurrently. IV drug use was the most common risk factor and found in 60% of cases [19]. 

Our patient had previously repaired mitral and tricuspid valve involvement and she remained an active intravenous abuser, which made her one of the high-risk individuals for getting community-acquired Serratia endocarditis.

In a study by Nikolakopoulos et al., Serratia is thought to induce resistance to cephalosporin by two distinct mechanisms: (i) Production of chromosomal AmpC cephalosporinases along with substantially decreased outer membrane permeability and (ii) β-lactamase production in order to hydrolyze carbapenems [4].

In a small retrospective analysis of 43 patients, 6 of whom had endocarditis with prosthetic valves, antibiotic regimens used for treatment were analyzed. As a part of antibiotic stewardship, the common idea is that Serratia induce AmpC beta-lactamases less commonly than ESBL; hence carbapenem and cefepime which are stable against Amp C beta-lactamases should be avoided as first line. The study observed that the most common treatment regimen was cefepime (44.2%) followed by meropenem (13.9%) or cefepime with aminoglycoside or fluoroquinolone (13.9%). Piperacillin-tazobactam was used in 11.6% of patients. 11.6% had mixed regimens based on switch between antibiotics. It was noted that a combination regimen was mostly used in endocarditis and cases where infectious disease was consulted. In 90.7% of cases, blood culture was negative within 48 hours of antibiotic start suggesting low pathogenicity. The patients with recurrences were treated with either a carbapenem or combination therapy. Seven patients died within 60 days of their first positive S. marcescens culture, attributable to comorbid conditions [2].

Our patient had been treated with meropenem due to resistance to piperacillin-tazobactam. Before discharge, her bacteremia had resolved temporarily, but due to severe widespread septic emboli seeding and devastating consequences, her clinical outcomes remained grim.

## Conclusions

Our patient was an unfortunate case based on her continued IV drug use and S. marcescens endocarditis on prosthetic valves. Given her embolic stroke, diffuse embolic phenomena, and instability for the surgery, her outcomes remained poor. 

This case also highlights the importance of abstinence from IV drug use, patient compliance and relapse rate, and lack of drug rehabilitation programs/ community resources. It is unsure if our patient sought any drug rehabilitation center or community source for her addiction.

We emphasize the continued importance of considering this organism in the differential diagnosis of endocarditis among IV drug users. This case also identifies candidates where surgery cannot be possible, although it can be the only potential cure. Management of these cases is always challenging. Combined efforts to treat underlying addiction are the key to preventing the reoccurrence of endocarditis in IV drug users.
